# Effects of an interactive mHealth innovation for early detection of patient-reported symptom distress with focus on participatory care: protocol for a study based on prospective, randomised, controlled trials in patients with prostate and breast cancer

**DOI:** 10.1186/s12885-017-3450-y

**Published:** 2017-07-04

**Authors:** Ann Langius-Eklöf, Marie-Therése Crafoord, Mats Christiansen, Maria Fjell, Kay Sundberg

**Affiliations:** Department of Neurobiology, Care Sciences and Society, Division of Nursing, Karolinska Institutet, 141 83 Huddinge, Stockholm, Sweden

**Keywords:** Information communications technology, mHealth, Application, Participatory care, Cancer supportive care, Cost-effectiveness, Study protocol, RCT, Clinical trial

## Abstract

**Background:**

Cancer patients are predominantly treated as out-patients and as they often experience difficult symptoms and side effects it is important to facilitate and improve patient-clinician communication to support symptom management and self-care. Although the number of projects within supportive cancer care evaluating mobile health is increasing, few evidence-based interventions are described in the literature and thus there is a need for good quality clinical studies with a randomised design and sufficient power to guide future implementations. An interactive information and communications technology platform, including a smartphone/computer tablet app for reporting symptoms during cancer treatment was created in collaboration with a company specialising in health care management. The aim of this paper is to evaluate the effects of using the platform for patients with breast cancer during neo adjuvant chemotherapy treatment and patients with locally advanced prostate cancer during curative radiotherapy treatment. The main hypothesis is that the use of the platform will improve clinical management, reduce costs, and promote safe and participatory care.

**Method:**

The study is a prospective, randomised, controlled trial for each patient group and it is based on repeated measurements. Patients are consecutively included and randomised. The intervention groups report symptoms via the app daily, during treatment and up to three weeks after end of treatment, as a complement to standard care. Patients in the control groups receive standard care alone. Outcomes targeted are symptom burden, quality of life, health literacy (capacity to understand and communicate health needs and promote healthy behaviours), disease progress and health care costs. Data will be collected before and after treatment by questionnaires, registers, medical records and biomarkers. Lastly, participants will be interviewed about participatory and meaningful care.

**Discussion:**

Results will generate knowledge to enhance understanding about how to develop person-centred care using mobile technology. Supporting patients’ involvement in their care to identify problems early, promotes more timely initiation of necessary treatment. This can benefit patients treated outside the hospital setting in regard to maintaining their safety.

**Clinical trial registration:**

June 12 2015 NCT02477137 (Prostate cancer**)** and June 12 2015 NCT02479607 (Breast cancer).

## Background

### Prostate and breast cancer patients’ needs

Prostate and breast cancer are currently among the most common cancer diagnoses worldwide [1] and the most common cancer diagnoses for men and women respectively in Sweden [2]. Due to developments in the treatment of prostate cancer, survival rates have improved [3, 4] but treatments come with a range of side effects, for instance urinary symptoms, bowel symptoms, pain, and fatigue, all of which affect patients’ quality of life (QoL) negatively [5–7]. Many of these symptoms can be long-lasting [7–9]. Likewise, advances in treatment have significantly improved breast cancer survival. Surgical excision of the tumour has generally been the first choice treatment but is increasingly being preceded by neo adjuvant systemic therapy. Similarly, this type of treatment regularly has side effects, including fatigue, dyspnoea, pain, nausea/vomiting, constipation and anxiety [10, 11], all of which impose substantial morbidity and burden on patients and their families and impact negatively on patients’ QoL, functioning and body image [12, 13].

Symptoms of cancer and side effects of treatment vary [14]; consequently, care and support should be based on the needs of the individual patient [7–9, 15]. Most patients in treatment remain living at home and this generally involves a degree of self-sufficiency in managing symptoms and side effects (self-care) including skills and knowledge concerning how to find and use information in regards to one’s health [16–18]. Currently, cancer patients may not receive adequate support to manage symptoms and side effects during treatment, resulting in a large number of patients visiting emergency departments, many of whom have to be hospitalised [19–23]. It has been concluded that self-care strategies are not a central focus for health care staff and patients [24–28] despite evidence that improvement in symptom management and self-care ability may lead to a faster return to daily activities and work [29, 30].

### Patient reported outcome measures (PROMs) and digitalization

Many cancer care providers have begun to incorporate patient-reported outcome measures (PROMs) into clinical practice, to support patients’ to be active in self-care and to identify when medical or nursing care interventions are needed [31–33]. A PROM includes any aspect of a patient’s health status (including disease symptoms, functioning and Health-related Quality of life-HRQoL) that is directly reported by the patient with no interpretation of the patient’s responses by a caregiver or anyone else [34]. Using PROMs in practice has been shown to improve patient-provider communication, facilitate early detection of problems, and to increase patient satisfaction [35–37]. For some time, it has been recognized that digital solutions can be of great assistance in these objectives [36, 38]. Therefore, evidence-based information and communication technology (ICT) which can contribute to early detection of symptoms and side effects within cancer care, is an urgent area for research, as this can aid prompt management, and increase patient safety and satisfaction.

### Mobile technology

Studies including technology for monitoring symptoms and providing self-care advice for cancer patients have been web-based [39, 40] or mobile -based [41]. The results show that interventions are user-friendly and feasible in clinical practice [41, 42] and increase patient-clinician communication [43]. The results also reveal effects, although small, on symptom management and symptom burden [40]. During the last decade there has been an increase in the number of scientific publications within the field of mobile health (mHealth) [44]. The vast majority concern the use of text messaging [44] but the use of apps is increasing, although research on its impact is scarce [45]. However, many apps focus on restricted aspects of the disease and hence risk failing to detect the multiple facets of the condition [45]. Moreover, apps have, among other issues, been criticized for lacking interactivity and for having content that is not relevant to users [44, 46]. Few studies exist on clinical outcomes and cost-effectiveness of using smartphone apps in health care [46].

### The development of an ICT platform - Interaktor

**Fig. 1:**
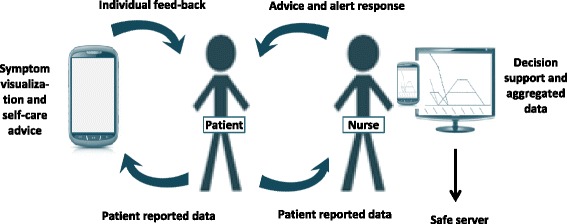
Illustration of the ICT-platform

We have developed an ICT platform with an interactive app that takes into account the different needs patients may experience as they manage symptoms and concerns related to an illness. The platform is developed as part of a formal cooperation between the research group and a Swedish Company, Health Navigator (HN) specialising in health care management. Interaktor includes a web interface and an app that is downloaded onto a smartphone or a tablet. The components are: 1) patients’ assessment of the occurrence, frequency and distress level of symptoms, 2) a web interface for the healthcare providers, for monitoring patients’ data in real time, 3) an alert function, based on a risk assessment model, which sends alerts to the nurses via text message (SMS), 4) access to evidence-based self-care advice related to symptoms and links to relevant websites, 5) graphs of symptom report history. The web interface functions both as an aid in patient-clinician communication about symptoms and self-care and as a decision aid for health care personnel managing symptoms. The reported data is stored at a designated secure server at HN (Fig. 1).

Development was guided by the framework of The Medical Research Council (MRC) [47] which recommend that complex interventions should be developed, tested and implemented in a process that encompasses three stepwise phases: i) defining and understanding the problem and the context; ii) developing the intervention and; iii) developing and optimizing the evaluation. The design and content of the app is based on the results of literature reviews and produced in a collaborative effort with patients and health care staff.

### Aim and hypothesis

This study aims to evaluate the effects of the interactive ICT platform (Interaktor) developed for this project, on patients with breast cancer during neo adjuvant chemotherapy treatment and patients with locally advanced prostate cancer during treatment with radiotherapy respectively. The main hypothesis is that the use of the platform will improve clinical management, reduce costs and promote safe and participatory care. Furthermore, the study population enables investigation of whether the intervention effects are generic. The specific research questions are:

1. Will using the app during treatment for breast and prostate cancer respectively:

- Minimize symptom burden?
- Enhance participatory and meaningful care?
- Improve the capacity to access, understand, communicate and use health information for health promoting behaviours?
- Impact the QoL positively, or affect disease progress and health care costs?

2. How does a person’s inner strength (sense of coherence) influence intervention effects?
3. How feasible, user-friendly and accepted is the platform according to patients and health professionals?
4. Are there any differences in study outcomes regarding diagnosis?

## Design and methods

The study has a prospective, repeated measure RCT design including patients with breast cancer during neo adjuvant chemotherapy treatment and patients with locally advanced prostate cancer during radiotherapy.

### Prostate cancer study

**Fig. 2:**
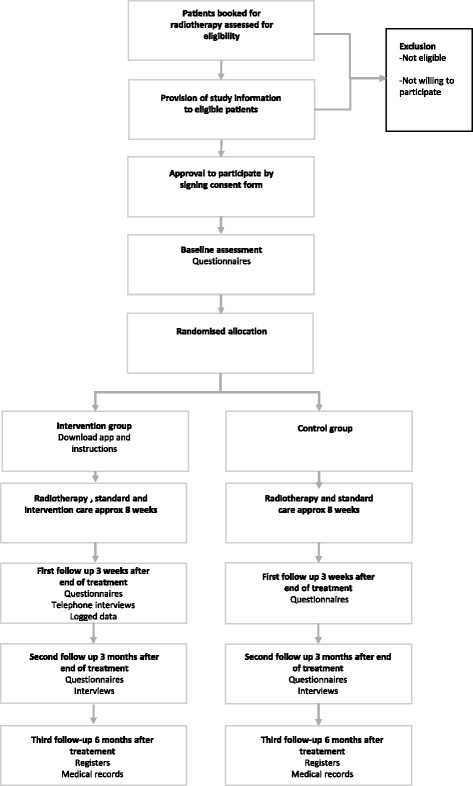
SPIRIT flow chart RCT-study prostate cancer

Data are collected during 20 weeks, at three time points; baseline, end of treatment and three months after the end of treatment, see SPIRIT study flow chart in Fig. 2. Patients are recruited from two clinics in Stockholm County Council in Sweden; the Department of Oncology at Karolinska University Hospital and the Department of Oncology Södersjukhuset. Recruitment began in August 2015 and completion for recruitment is expected in August 2017. A sample of 150 prostate cancer patients will be recruited. Inclusion criteria are; patients diagnosed with prostate cancer, scheduled to receive curative radiotherapy for at least five weeks. Exclusion criteria are: inability to read or understand Swedish, or having a known severe cognitive disability. Patients scheduled for radiotherapy are consecutively identified for eligibility by one of the researchers by screening of the booking lists. Eligible patients are sent a letter with information about the study and contacted by one of the researchers via telephone approximately a week before their first treatment appointment. Those who agree to participate sign a consent form and fill out the baseline assessment questionnaire before being assigned to either the intervention or control group by a sequence of sealed envelopes with equal distribution among the two groups [48].

#### Intervention and standard care

All patients receive standard treatment and care according to clinical guidelines and the protocol of the clinic where they are treated. This includes radiotherapy five days a week, with or without a combination of two sequences of brachytherapy, and regular contact with an assigned oncology contact nurse who can be contacted during office hours. The patients in the intervention group download the app onto their own smartphone or tablet; alternatively they may borrow such a device from the research group. They are instructed on how to use the app for reporting symptoms and start reporting in the app on their first day of radiotherapy. They then report daily, during weekdays, and three weeks following the last treatment. The total time for reporting is between eight to 11 weeks depending on whether the treatment is a combination treatment or not. The nurses at the clinic receive instructions and training on how to use the platform, including the alert system, and how to monitor the patients´ reports. Reporting in the app is a complement to the usual care and patients are encouraged to report before 3 PM as reports and alerts are managed during office hours (7 AM-4 PM). If emergency health care attention is needed the patients are instructed to contact the health care according to the standard procedure at the oncological clinic.

#### The prostate cancer version of Interaktor

The prostate cancer version includes 14 symptom questions regarding bladder and bowel function, fatigue, pain, worry, depression, sleep, and flushing. They are included as a result of a literature review, interviews with patients and healthcare providers [25] and a feasibility study [49]. Furthermore, there is an open question, providing the patient with an opportunity to add comments; *“Other symptoms or concerns to report?”* Patients are asked about the symptoms’ occurrence, frequency, and distress level during the day, for example: *“Do you experience urinary difficulties?”* If the answer is yes, the patient is asked about the frequency, which can be rated as: almost never, sometimes, rather often, or very often. Next follows a question on distress level, which can be rated as: not at all, a little, somewhat, or very much. The symptoms of fatigue, insomnia, constipation and blood in stool are only assessed by the distress level and not by frequency. Specific symptoms are set to generate an alert to the registered nurses, via text messages (SMS), on appointed levels of frequency or distress. The level for each symptom has been decided based on a risk assessment model, in collaboration with clinicians. There are two kinds of alerts: yellow alerts that request a nurse to contact the patient during the day, and red alerts, requiring contact within one hour. Severe symptoms regarded as a potential risk for the patients’ health and well-being trigger a red alert. A symptom considered to be less severe triggers a yellow alert. The symptoms set to trigger alerts are: urinary urgency (very often; yellow alert), difficulties urinating (very often; red alert), haematuria (very often; yellow alert), diarrhoea (very often; yellow alert), blood in stool (very much; red alert), obstipation (very much; yellow alert), pain (very often; red alert) and depression, worry (very often; red alert).

### Breast cancer study

**Fig. 3:**
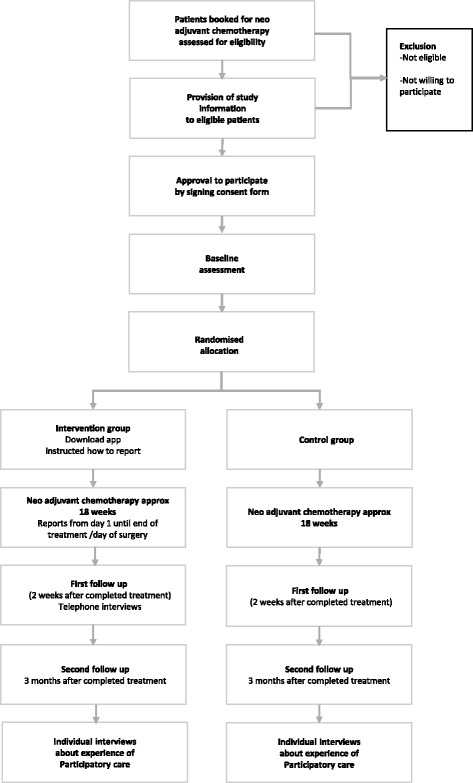
SPIRIT flow chart RCT-study breast cancer

Data are collected over 30 weeks, at three time points; baseline, end of treatment and three months after the end of treatment, see SPIRIT study flow chart in Fig. 3. Patients are recruited from two clinics in Stockholm County Council in Sweden; the Department of Oncology at Karolinska University Hospital and the Department of Oncology Södersjukhuset. Recruitment began in June 2015 and is expected to be complete in June 2017. A sample of 150 breast cancer patients will be recruited. Inclusion criteria are: patients with breast cancer receiving neo adjuvant chemotherapy, men or women, 18 years or older. Exclusion criteria are: unable to read and understand Swedish, or patients with a known severe cognitive disability. Eligible patients are consecutively identified by one of the researchers through screening of booking lists and are provided with written information by the assigned oncology nurse or physician during their first visit. The patients who approve to be contacted by the researcher are called and a meeting is arranged. The patients consent to participate in the study by signing a consent form and after that fill in the baseline assessment questionnaire before being randomised to either the intervention or the control group by a sequence of sealed envelopes with equal distribution among the two groups [48].

#### Intervention and standard care

All patients receive standard treatment and care according to clinical guidelines and the protocol of the clinic where they are treated. Standard treatment and care consist of neo adjuvant chemotherapy and regular visits to the physician and the oncology contact nurse prior to every treatment occasion. The patients in the intervention group download the app onto their own smartphone or tablet; alternatively they may borrow such a device from the research group. They are instructed on how to use the app for reporting symptoms and start reporting in the app on their first day of chemotherapy. They then report daily, during weekdays, and two weeks following the last treatment, alternatively until the day of surgery. The total reporting time is approximately 18 weeks. Reporting in the app is complementary to the usual care and patients are encouraged to report before 3 PM as the reports and alerts are managed during office hours (7 AM- 4 PM). If emergency health care attention is needed the patients are instructed to contact the health care according to the standard procedure at the oncological clinic.

#### The breast cancer version of Interaktor

The breast cancer version includes 14 symptom questions regarding fever, breathing difficulties, pain, nausea, vomiting, bowel function, oral problems, worry, depression, fatigue, insomnia, numbness/tingling in the hands and feet, and pain/swelling/redness in the arm (relating to the peripherally inserted central catheter line for chemotherapy administration). Furthermore, there is an open question providing the patient with an opportunity to add comments; *“Other symptoms or concerns to report?”* The questions were formulated based on extant literature and guidelines and subsequently pilot-tested on eight patients. Results from the feasibility study of the prostate cancer version were considered sufficient and thus no feasibility study was conducted for the breast cancer version. Patients are asked about the symptoms’ occurrence, frequency, and distress level during the day, for example: *“Do you experience nausea?”* If the answer is yes, the patient is asked about the frequency, which can be rated as: never, sometimes, rather often, or very often. Next follows a question on distress level, which can be rated as: not at all, a little, somewhat, or very much. The symptoms fever and pain/swelling/redness in the arm are only assessed by occurrence. The symptoms constipation, oral problems, worry, distress and insomnia are only assessed by the distress level and not frequency. Specific symptoms are set to generate an alert to the registered nurses via text messages (SMS) triggered by either occurrence, frequency or distress level. The level for each symptom was decided based on a risk assessment model, in collaboration with clinicians. There are two kinds of alerts: yellow alerts that request a nurse to contact the patient during the day, and red alerts, requiring contact within one hour. Severe symptoms regarded as a potential risk for the patients’ health and well-being trigger a red alert. A symptom considered to be less severe generates a yellow alert. The symptoms set to generate alerts are: fever (yes; red alert), pain/swelling/redness in the arm (yes; yellow alert) breathing difficulties, nausea and vomiting (rather often; yellow alert, very often; red alert) diarrhoea (very often; yellow alert), obstipation and worry (very distressing; yellow alert) and lastly oral problems (somewhat distressing; yellow alert).

### Primary outcomes

#### Questionnaires

Outcomes concerning HRQoL, symptom distress, perception of individual care, sense of coherence and health literacy will be collected through validated self-report questionnaires;

- EORTC-QLC-C30 (including disease-specific module PR-25 in the prostate study) for the evaluation of HRQoL [50].
- The Memorial Symptom Assessment Scale (MSAS) a 32-item questionnaire for measuring symptom prevalence, characteristics and distress (Breast cancer study only) [51].
- The Individual Care scale (ICS): a 24-item self-measurement of perception of individual care [52].
- The Sense of Coherence Scale a 13-item questionnaire for measuring overall coping ability (inner strength) [53, 54].
- The Health literacy scale: a ten-item questionnaire assessing functional, communicative and critical health literacy [55].

#### Registers and logged data

Medical data (morbidity, mortality, recurrence rate, biomarkers) will be obtained from medical journals, and socioeconomic data and health care costs (visits, pharmaceuticals) will be collected from medical registers up to six months after the intervention for a cost-effectiveness analysis of the intervention. Logged data on symptom reports and self-care advice accessed (adherence to the intervention) including frequencies of symptoms and generated alerts will be collected.

#### Interviews about feasibility and acceptability

On to two weeks following the intervention all the patients in the intervention group are interviewed via telephone. Interviews are based on a semi-structured interview guide exploring the feasibility and acceptability of the app, the content of the self-care advice, and technical or other problems encountered when using the system.

#### Interviews about participatory and meaningful care

Eighty patients (40 with breast cancer and 40 with prostate cancer) from both the intervention groups and the control groups will be interviewed face to face and individually about their experiences of participatory and meaningful care. The focus will be to explore how patients perceive their life situation in relation to the disease and how involved they felt in their care during the treatment. The interviews will be conducted three months after the end of treatment.

Health care professionals involved in the care of the patients in the intervention group will be interviewed via focus groups about their perceptions of the overall use of the mobile phone system and how the intervention might enhance participatory care. Special focus will be on symptom assessment and care interventions initiated because of the alert system, transfer of information and generation of alerts, and lastly the content and delivery of the self-care advice. We believe that the interaction between health care professionals in focus group interviews will give deep insights into the complexity of this phenomenon.

### Sample size calculation

The sample size is estimated from the results of the effect study including patients with prostate cancer, Sundberg et al. [56] With an effect size (Cohen’s d) difference of 0.54 in the primary outcome (urinary symptom) with 90% power at *p* < 0.05, 71 patients in each group are needed. A similar study with an expected effect size (Cohen’s d) difference of 0.20 in the primary outcome (symptom improvement) with 85% power at *p* < 0.05 with five repeated measurement needed 133 patients in each group [40]

### Statistical analysis

Data will be analysed using the IBM SPSS statistical software package (version 24.0.) Analysis will be performed according to the intention-to-treat, ITT principle, and the main aim will be to examine differences between and within groups and to investigate independent variables that may explain the outcome. The analysis will include standard descriptive statistics, and inferential analyses based on linear mixed-effect models accounting for repeated measurements [57].

Furthermore, latent class analysis will be used for each person and across the group of patients to identify groups that share characteristics with different components related to the intervention and its outcome [58]. Analyses will be performed in collaboration with a consultant statistician and a health-economist.

### Qualitative analysis

Interviews with patients and health care staff will be transcribed verbatim and analysed through inductive and deductive qualitative content analysis according to Elo and Kyngäs [59].

## Discussion

For some time now, mHealth has been predicted to be a future disruptive innovation which may revolutionise the way health care is organised [60–63]. The development and implementation of digital solutions have been encouraged by policymakers as they have been anticipated to decrease the rising health care costs which are due to an ageing population and technical advances in treatments [60, 62]. However, a plethora of articles are concerned with why the vast majority of eHealth, tele health or mHealth projects fail [45, 64]. Authors have called for studies which can provide solid and generalizable results, as well as studies considering context, values and more qualitative aspects of health technology evaluation and implementation [65–67].

Studies on clinical outcomes and cost-effectiveness of apps are sparse [46]. Since the launch of this study, promising results have emerged indicating that mHealth can have a positive effect on cost-effectiveness as well as patient safety. Basch [68] found that monitoring PROM via a computer tablet during cancer treatment improved HRQoL and resulted in fewer admissions to an emergency room or hospitalisations compared to the control group. This suggests that mHealth could function as a cost-effective method to promote communication between patients and clinicians, and to enable health care staff to tailor the care to the individual patient’s needs. In a study by Drott [69] the results showed that reporting side effects during treatment for colorectal cancer via a mobile phone based system reinforced the patient’s experience of being involved in their care.

The results of this study will provide helpful knowledge and insights into the effects of using an app for monitoring and managing symptoms and side effects during cancer treatment in a population consisting of both breast and prostate cancer patients. The size and characteristics of the cohorts will allow inferences on differences in effect between sex/diagnosis to be drawn, factors which have been found to influence attitudes and usage of mobile health technologies [70–73].

## Abbreviations

EORTC QLQ-C30: European Organisation for Research and Treatment of Cancer Core Quality-of-Life
HRQL: Health-Related Quality of Life
ICT: Information Communications Technology
ITT: Intention-to-treat
MRC: Medical Research Council
MSAS: Memorial Symptom Assessment Scale
PROM: Patient Reported Outcome Measures
QoL: Quality of Life
SOC: Sense of Coherence
